# Impact of physiological noise correction on detecting blood oxygenation level-dependent contrast in the breast

**DOI:** 10.1088/1361-6560/62/1/127

**Published:** 2016-12-13

**Authors:** Tess E Wallace, Roido Manavaki, Martin J Graves, Andrew J Patterson, Fiona J Gilbert

**Affiliations:** 1 Department of Radiology, University of Cambridge, Cambridge Biomedical Campus, Cambridge, UK; 2 NIHR Cambridge Biomedical Research Centre, Cambridge Biomedical Campus, Cambridge, UK; 3 Department of Radiology, Cambridge University Hospitals NHS Foundation Trust, Cambridge, UK; fjg28@medschl.cam.ac.uk

**Keywords:** functional magnetic resonance imaging, physiological noise, retrospective image correction, BOLD contrast, haemodynamic response

## Abstract

Physiological fluctuations are expected to be a dominant source of noise in blood oxygenation level-dependent (BOLD) magnetic resonance imaging (MRI) experiments to assess tumour oxygenation and angiogenesis. This work investigates the impact of various physiological noise regressors: retrospective image correction (RETROICOR), heart rate (HR) and respiratory volume per unit time (RVT), on signal variance and the detection of BOLD contrast in the breast in response to a modulated respiratory stimulus. BOLD MRI was performed at 3 T in ten volunteers at rest and during cycles of oxygen and carbogen gas breathing. RETROICOR was optimized using *F*-tests to determine which cardiac and respiratory phase terms accounted for a significant amount of signal variance. A nested regression analysis was performed to assess the effect of RETROICOR, HR and RVT on the model fit residuals, temporal signal-to-noise ratio, and BOLD activation parameters. The optimized RETROICOR model accounted for the largest amount of signal variance ($ \Delta R_{\text{adj}}^{2}$  =  3.3  ±  2.1%) and improved the detection of BOLD activation (*P*  =  0.002). Inclusion of HR and RVT regressors explained additional signal variance, but had a negative impact on activation parameter estimation (*P*  <  0.001). Fluctuations in HR and RVT appeared to be correlated with the stimulus and may contribute to apparent BOLD signal reactivity.

## 1. Introduction

Blood oxygenation level-dependent (BOLD) contrast exploits the differential magnetic properties of oxygenated and deoxygenated haemoglobin to enable detection of changes in blood oxygenation and flow. BOLD contrast is extensively used in functional magnetic resonance imaging (fMRI) experiments to map brain activation in response to a stimulus or to depict resting-state functional connectivity. As BOLD signal changes are small, they require a high temporal signal-to-noise ratio (TSNR) for reliable detection (Murphy *et al* 2007). Imaging at higher static magnetic field strengths (*B*_0_) increases SNR and hence TSNR; however, the relative contribution of physiological noise also increases with field strength, thereby decreasing signal detection power (Kruger and Glover 2001). Several sources of physiological noise have been identified in the fMRI literature, including those associated with cardiac (Dagli *et al* 1999, Shmueli *et al* 2007) and respiratory (Wise *et al* 2004, Birn *et al* 2006) processes, and residual movement artefacts after registration (Lund *et al* 2005).

There is growing interest in applying BOLD contrast outside of the brain to assess tumour oxygenation and angiogenesis via vasomotor response to modulated hyperoxic and hypercapnic gas stimuli. These experiments are analogous to fMRI of the brain, with the exception that a respiratory stimulus is needed to directly modulate blood oxygenation and flow. BOLD MRI has been used to demonstrate improved oxygenation in response to inhalation of 100% oxygen or carbogen (2–5% CO_2_; 95–98% O_2_) in a variety of solid tumour types, including breast cancer, to derive potential markers of tumour hypoxia (Griffiths *et al* 1997, Taylor *et al* 2001, Rijpkema *et al* 2002, Alonzi *et al* 2009, Jiang *et al* 2013). A pilot study in breast cancer patients undergoing neoadjuvant chemotherapy (*n*  =  7) demonstrated that oxygen-induced BOLD contrast changes were significantly greater (*P*  <  0.001) in patients exhibiting a complete pathological response versus those exhibiting partial response or stable disease (Jiang *et al* 2013). These studies suggest that BOLD MRI may aid patient stratification for hypoxia-targeted therapies and has potential to provide early predictive response monitoring. Other studies in the breast have shown that 100% oxygen interleaved with carbogen (5% CO_2_, 95% O_2_) in a block design is the optimal stimulus for inducing BOLD contrast (Rakow-Penner *et al* 2010, Wallace *et al* 2016a). Carbon dioxide is a potent vasodilator and the opposing effects of these two gases on vascular tone provide a mechanism for BOLD contrast, which is also sensitive to changes in blood volume and flow. In theory, healthy vasculature will constrict and dilate in response to vasoactive stimuli, but immature tumour vessels lacking appropriate smooth muscle vasculature will be unable to respond. Several studies in preclinical tumour models have demonstrated the potential of BOLD contrast as a functional biomarker of vascular maturity (Neeman *et al* 2001, Gilad *et al* 2005) and this approach has been successfully translated to derive a functional vascular maturation index in human brain tumours (Ben Bashat *et al* 2012). However, optical imaging studies have suggested that in a clinical setting physiological fluctuations may confound measurement of haemodynamic response (Carpenter *et al* 2010a, 2010b). Respiration leads to both motion artefacts and modulation of the magnetic field, and is expected to be a particularly significant source of noise, depending on the target site. In general, background physiological variations and motion increase signal variance, and may even give rise to false-positive activation effects if they happen to be correlated with the stimulus.

Various methods for reducing physiological noise have been proposed, operating both in *k*-space (Hu *et al* 1995, Le and Hu 1996, Wowk *et al* 1997) and image space (Glover *et al* 2000, Deckers *et al* 2006). Many correction schemes require acquisition of additional physiological data using peripheral measures of cardiac and respiratory function (Hu *et al* 1995, Glover *et al* 2000), whilst a few methods utilize the MRI data itself to estimate noise parameters (Le and Hu 1996, Wowk *et al* 1997). Most physiological noise models can be included as nuisance variables in a general linear model (GLM) regression analysis (Lund *et al* 2006, Shmueli *et al* 2007, Kong *et al* 2012), although some may be used for straightforward data correction (Glover *et al* 2000). RETROICOR is an established retrospective image-based correction method, which is frequently applied in brain fMRI experiments to improve the statistical significance of activation signals. RETROICOR models cardiac and respiratory fluctuations using a Fourier series defined by the phase of the cardiac and respiratory cycles, relative to the time of image acquisition. The standard RETROICOR implementation, optimized for the cerebrum, comprises two respiratory and two cardiac harmonics (Glover *et al* 2000). Harvey *et al* (2008) implemented a modified version of the RETROICOR algorithm to include higher order and multiplicative terms to account for the interaction between cardiac and respiratory signals. The optimized RETROICOR model significantly reduced signal variability in the brainstem and improved detection of activation in response to a finger-tapping task (Harvey *et al* 2008).

As well as quasi-periodic physiological fluctuations, low frequency (<0.1 Hz) variations related to respiratory depth and rate have previously been correlated with changes in BOLD signal intensity. Studies have shown that subtle fluctuations in respiratory volume per unit time (RVT) can account for a significant amount of variance in the resting-state BOLD signal (Wise *et al* 2004, Birn *et al* 2006). Shmueli *et al* (2007) additionally found significant correlations between heart rate (HR) and BOLD signal time courses. The authors demonstrated that including delayed HR time series regressors in a GLM was able to explain an additional 1% of BOLD signal variance, beyond that explained by RVT and RETROICOR regressors (Shmueli *et al* 2007). Both cardiac (Chang *et al* 2009) and respiratory (Birn *et al* 2006) response functions have been proposed in the brain fMRI literature to model these physiological noise effects. Decreasing signal variance should theoretically improve BOLD sensitivity. Hutton *et al* (2011) found that applying a combination of physiological noise correction models, including HR and RVT regressors, resulted in a 50–70% increase in TSNR, which translated to a 10% increase in the number of significantly activated voxels in fMRI (Hutton *et al* 2011).

The impact of these physiological correction techniques on BOLD signal variance and sensitivity has been investigated in the fMRI literature; however, their use outside the brain has been limited. Cardiac and respiratory fluctuations, including low frequency variations in respiratory volume and heart rate, are expected to influence BOLD sensitivity and parameter estimation. The purpose of this work was to identify and remove variance in the BOLD signal attributed to physiological noise sources in order to improve detection of vascular reactivity to CO_2_. We sought to optimize the RETROICOR algorithm to account for the maximum amount of signal variance, without over-fitting to noise. We also assessed the impact of the optimized RETROICOR algorithm and regression of HR and RVT on the detection of haemodynamic response via BOLD contrast in the breast, both at rest and in response to a modulated respiratory stimulus paradigm.

## 2. Methods

### 2.1. Subjects and stimulus design

**Figure 1.**
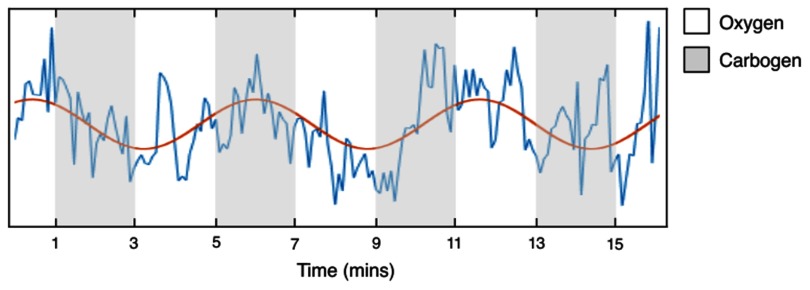
Schematic showing the modulated gas fMRI stimulus design. Oxygen and carbogen are cycled in 2 min blocks for a total of 16 min. An example BOLD signal intensity time course extracted from the fibroglandular tissue in a representative volunteer is shown, with the sinusoidal model used to fit the signal intensity response (phase shifted to match the time lag of response and scaled to the amplitude of response) overlaid in red.

MRI data was collected from ten healthy female volunteers aged between 22 and 37 years (median age 27 years). All participants provided written informed consent prior to enrolment in the study, which was approved by the local Research Ethics Committee (REC: 14/EE/0145). Resting state data was acquired as subjects breathed medical air for 12 min. The respiratory gas stimulus paradigm consisted of breathing carbogen interleaved with 100% oxygen in 2 min blocks, for a total of 16 min, as illustrated in figure 1. Medical gases were administered to the subject via an OxyMask^™^ (Southmedic Inc., Barrie, ON) at a flow rate of 14 l min^−1^, with automated switching controlled by an in-house gas delivery system.

### 2.2. Data acquisition

MR imaging was performed at 3 T (MR750, GE Healthcare, Waukesha, WI) using an eight-channel phased-array breast coil with whole body radiofrequency excitation. An in-house developed multi-phase single-shot fast spin echo sequence was used to acquire dynamic *T*_2_-weighted images at a single sagittal slice location. A *T*_2_-weighted spin-echo based sequence was used for BOLD contrast generation, rather than the more conventional $T_{\text{2}}^{\ast}$-weighted gradient echo imaging, due to the adverse *B*_0_ field distortions created by the breast geometry at higher field strengths (Rakow-Penner *et al* 2010). Scan parameters were as follows: TR  =  4000 ms, effective TE  =  58 ms, ±83 kHz receiver bandwidth, 128 phase and frequency encoding steps, 20 cm field of view, 5 mm slice thickness and chemical shift selective fat suppression. A total of 180 and 240 images were acquired for the resting-state and oxygen-carbogen datasets respectively, with an in-plane spatial resolution of 1.56  ×  1.56 mm. A high-resolution *T*_1_-weighted image was acquired at the same sagittal slice location to provide anatomical detail.

Heart rate and respiration were monitored using the scanner’s built-in photoplethysmograph placed on the subject’s index finger and a pneumatic respiratory belt positioned around the abdomen. The cardiac pulse signal and respiratory waveform were sampled at 40 Hz and 10 Hz respectively, and recording was triggered by the start of the scan acquisition. Timing data for each slice acquisition was written to a file to allow retrospective synchronization of the physiological signals with the MRI data.

### 2.3. Image registration and data pre-processing

Each image series was registered using a non-rigid symmetric diffeomorphic normalization (SyN) based algorithm (Advanced Normalization Tools, Philadelphia, PA) to compensate for patient motion during the MR acquisition (Avants *et al* 2011). Mutual information was used as the similarity metric and all phases were registered to the mean of the time series. A constrained cost-function masking approach was used to localize the motion correction to the breast region. The non-rigid registration was initialized with an affine transform to account for any global motion in the breast. A multi-resolution framework (four resolution levels) was used for both the affine and SyN registration.

All subsequent data processing was performed using in-house software developed in Matlab version 8.6 (The Mathworks, Natick, MA). For the scans where subjects breathed the oxygen-carbogen stimulus, imaging data from the first alternating gas period (i.e. first 60 time points) was discarded to allow equilibration of the gas inhalation regime. A region of interest (ROI) was manually drawn to exclude fat in the outer border of the breast, and subsequent analysis was performed pixel-wise within this fibroglandular tissue ROI in each volunteer. A second order polynomial regressor was constructed by fitting linear and quadratic functions to the signal intensity-time course for each pixel to model low frequency temporal drifts, nominally attributed to the scanner hardware.

### 2.4. Physiological noise regression models

The registered datasets were corrected for cardiac pulsatility and respiratory motion artefacts using a modified version of the RETROICOR algorithm. *F*-test regression analysis was performed to compare models with different combinations of cardiac (C), respiratory (R) and multiplicative (X) terms to find the combination that most effectively reduced BOLD signal variability without over-fitting to noise. The construction and optimization of the RETROICOR model is described in detail in the appendix.

Low frequency changes in respiratory volume per unit time (RVT) and heart rate (HR) were modelled using previously published methods (Birn *et al* 2006, Shmueli *et al* 2007) (see appendix). The HR and RVT regressors were created by shifting the HR and RVT time courses relative to the imaging data, based on the time lag that resulted in the maximum correlation between HR and RVT and the BOLD signal in each pixel (Birn *et al* 2006). The temporal cross correlation was calculated pixel-wise over the range  ±1 min (±15 TR), which yielded normalized correlation coefficients for each temporal shift of HR and RVT, relative to the BOLD signal.

The mean and standard deviation of HR and RVT, as well as the correlations between the two time courses (within  ±1 min), were calculated for each subject at rest and during cycling of oxygen and carbogen. Fourier power spectra of the HR and RVT time courses were also computed for each subject both at resting-state and for the respiratory stimulus.

### 2.5. Model evaluation

To evaluate the effect of different physiological noise models, a nested regression analysis was performed using the GLM framework in Matlab. Five models were defined as follows: (1) HW, (2) HW  +  CRX, (3) HW  +  CRX  +  HR, (4) HW  +  CRX  +  RVT, (5) HW  +  CRX  +  HR  +  RVT, where HW is the second order polynomial regressor constructed to account for low frequency hardware drifts, CRX represents the optimized RETROICOR regressor combination, and HR and RVT regressors are computed as outlined above. The regressors were fitted to the registered image data (following mean correction) using a GLM and correction was performed by subtracting the fitted effect from the BOLD signal.

The adjusted coefficient of determination ($R_{\text{adj}}^{2}$) was calculated as a measure of the proportion of variance accounted for by each of the models, normalized by the number of regressors. The regression models were chosen such that the explanatory power of each individual correction could be determined by comparing the results of different models. Pixel-wise $ \Delta R_{\text{adj}}^{2}$ values were calculated by subtraction of different models as a measure of the additional variance accounted for by each set of regressors. For example, comparing Models 1 and 2 ($ \Delta R_{\text{adj}\left(\text{2-1}\right)}^{2}$) gives an indication of how much additional variance is explained by the RETROICOR regressors (CRX) alone. Maps of $R_{\text{adj}}^{2}$ and $ \Delta R_{\text{adj}}^{2}$ were created for each subject and values were averaged within the fibroglandular tissue ROI and across subjects.

We also investigated the impact of each of the physiological noise models on the TSNR of the imaging data. TSNR was calculated as the mean of each pixel divided by the standard deviation of the time course. Maps of TSNR and percentage improvement in TSNR were created for each subject and the mean TSNR and ΔTSNR (%) were calculated within the fibroglandular tissue ROI and averaged across subjects. No spatial smoothing was applied to the datasets in this analysis, consistent with previous investigations of the impact of physiological noise regressors on $R_{\text{adj}}^{2}$ and TSNR (Kruger and Glover 2001, Hutton *et al* 2011).

### 2.6. Impact on BOLD parameter estimation

Prior to functional parameter estimation, both the resting-state and oxygen-carbogen datasets were smoothed using a Gaussian kernel (full width half maximum 4 mm), as spatial filtering is a commonly employed pre-processing step in these types of analyses (Wise *et al* 2004, Harvey *et al* 2008, Hutton *et al* 2011). The linear correlation coefficient between each pixel’s signal intensity-time course and sine and cosine functions at the stimulus frequency (0.0042 Hz) was calculated as a measure of the magnitude of the BOLD response, as described by Lee *et al* (1995). A sinusoidal waveform was chosen to model the block design stimulus as the haemodynamic response function effectively acts as a temporal low pass filter on the time series (Bulte *et al* 2006). The cosine function accounts for unknown delays in response. The magnitude of the maximum correlation coefficient and the temporal phase lag at which it occurs are given by the following expressions:

1 \begin{eqnarray*}{{r}_{\text{m}}}=\sqrt{r_{\text{s}}^{2}+r_{\text{c}}^{2}}\end{eqnarray*}

2 \begin{eqnarray*}{{\theta}_{\text{r}}}={{\tan}^{-1}}\left(\frac{{{r}_{\text{s}}}}{{{r}_{\text{c}}}}\right)\end{eqnarray*}

where *r*_s_ and *r*_c_ are the linear correlation coefficients between the BOLD signal intensity response and the sine and cosine waveforms, *r*_m_ ranges from 0 to 1 and *θ*_r_ ranges from 0 to 2*π*.

This cross-correlation analysis with the stimulus was performed following each model correction (i.e. subtraction of the fitted regressors from the BOLD signal time course). The same analysis was carried out for the resting-state data, even though there was no imposed stimulus periodicity, to determine the impact of each model correction on the extent of false-positive activation effects. Significantly activated pixels were defined using an uncorrected *p*-value threshold of less than 0.05 (*r*_m_  >  0.14) and the percentage of activated pixels within the fibroglandular tissue ROI was calculated for each subject during rest and activation. The median *r*_m_ was also calculated over all pixels within the fibroglandular tissue ROI to allow unbiased comparison of oxygen-carbogen and resting-state data.

### 2.7. Statistical analysis

Two-tailed, paired (across subjects) Student’s *t*-tests were performed to assess the impact of each correction on the TSNR, median correlation coefficient (*r*_m_) and percentage of activated pixels in resting-state scans. Paired *t*-tests were also performed to assess the effect of each correction on the median *r*_m_ and percentage of activated pixels in response to the vasoactive stimulus, relative to air-only breathing.

## 3. Results

### 3.1. Low frequency HR and RVT fluctuations

**Figure 2.**
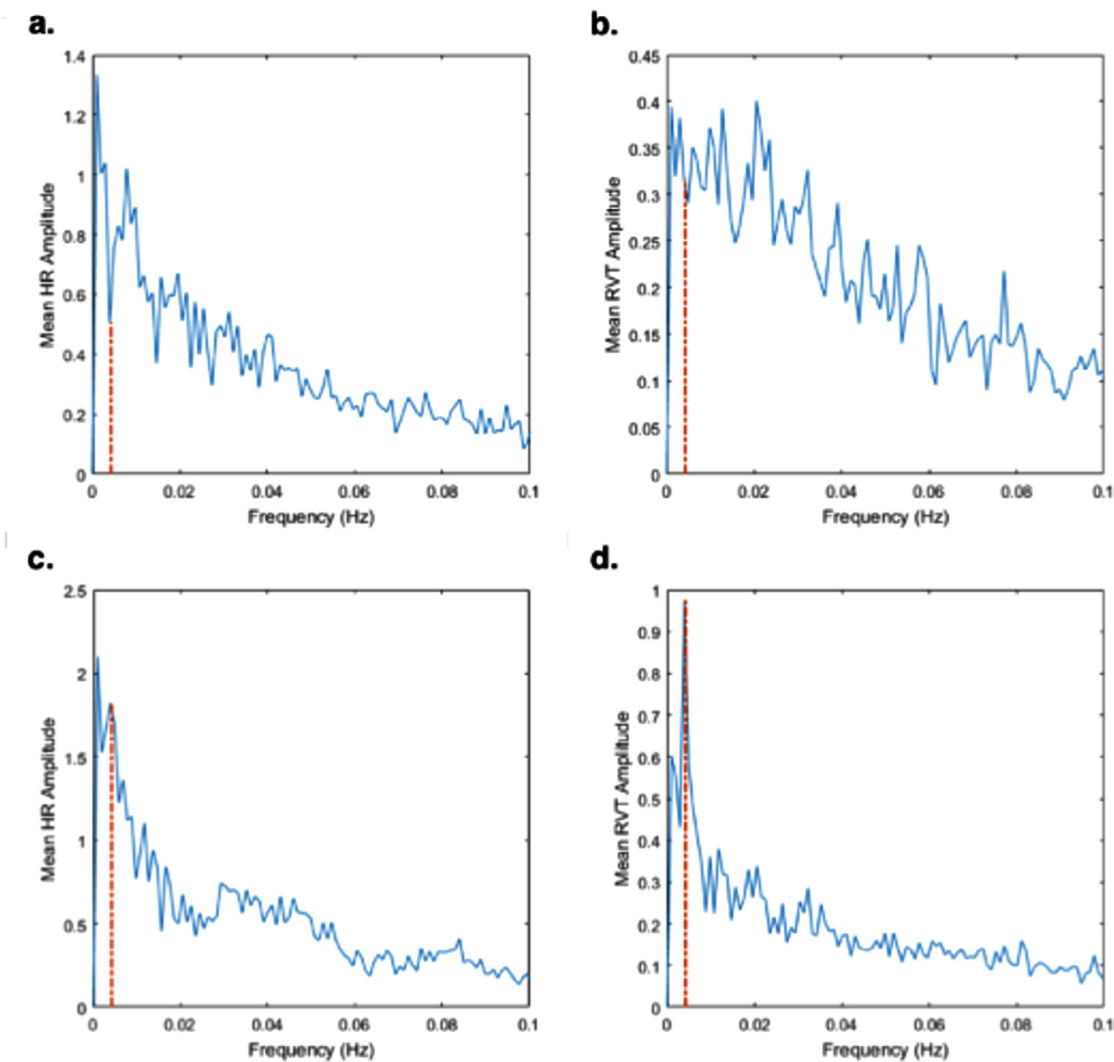
Fourier power spectra of HR and RVT time courses during (a) and (b) air-only breathing and (c) and (d) oxygen-carbogen breathing averaged over all subjects. The red dashed line denotes the stimulus frequency (0.0042 Hz).

Summary statistics for HR and RVT, and the correlation between these two physiological time courses, are shown for each subject in table 1. One subject was excluded, due to failure to record a reliable physiological trace. The mean HR across all subjects was 70.2  ±  3.1 beats min^−1^ (bpm) during air-only breathing and 68.8  ±  4.3 bpm during cycling of oxygen and carbogen gas. RVT fluctuated about 8.2  ±  1.6% at rest and about 9.2  ±  1.6% during oxygen-carbogen breathing. Resting-state HR and RVT were moderately correlated (*R*  =  −0.323 to 0.403), and the strength of the correlation increased for interleaved oxygen and carbogen breathing in some subjects (*R*  =  −0.290 to 0.650). Figure 2 shows the Fourier power spectra of HR and RVT both at rest and during the modulated respiratory stimulus. This confirms that fluctuations in heart rate and respiration occur in the low (<0.1 Hz) frequency range. Both HR and RVT spectra exhibited a peak at the stimulus frequency (0.0042 Hz) during oxygen-carbogen breathing, suggesting they are correlated with the stimulus paradigm.

**Table 1.** Mean and standard deviation (SD) of measured heart rate (HR) and respiratory volume per unit time (RVT) in each subject and the maximum cross correlation coefficient (CC) between HR and RVT for both air-only and modulated oxygen-carbogen breathing.

	Air-only			Oxygen-carbogen		
Subject	HR^a^ (bpm)	RVT^b^	CC^c^	HR (bpm)	RVT	CC
1	75.1 ± 1.7	5.7 ± 0.9	−0.323	70.9 ± 2.1	6.9 ± 1.2	0.508
2	70.1 ± 2.8	5.7 ± 0.7	0.316	62.3 ± 2.1	5.6 ± 0.8	−0.246
3	57.6 ± 6.0	11.1 ± 1.7	−0.251	59.0 ± 7.6	11.4 ± 1.4	−0.283
4	59.3 ± 3.1	9.5 ± 1.9	0.219	58.7 ± 2.9	10.4 ± 1.8	0.243
5	86.2 ± 3.0	11.5 ± 2.1	−0.207	86.3 ± 12.2	10.8 ± 1.5	−0.151
6	77.9 ± 3.1	6.8 ± 1.5	−0.176	79.0 ± 3.3	8.6 ± 1.4	−0.290
7	74.2 ± 3.1	9.2 ± 1.2	0.246	74.8 ± 3.1	11.1 ± 1.4	0.276
8	69.8 ± 2.2	6.8 ± 2.6	0.328	67.4 ± 2.6	9.7 ± 2.9	0.650
9	60.7 ± 2.7	7.4 ± 1.5	0.403	61.0 ± 3.1	9.0 ± 2.2	0.624
Mean	70.2 ± 3.1	8.2 ± 1.6		68.8 ± 4.3	9.2 ± 1.6	
SD	9.5 ± 1.2	2.2 ± 0.6		9.7 ± 3.4	2.0 ± 0.6	

a Heart rate.

b Respiratory volume (in percent) per unit time (s).

c Maximum correlation coefficient.

### 3.2. Optimization of RETROICOR

*F*-test results in table 2 show the percentage of all pixels in which each test model accounted for significantly more variance than the base model containing a subset of its regressors. Numbers in bold correspond to values above the percentage of pixels set by a binomial (null) threshold. The first order respiratory regressor (1R) accounted for the largest amount of noise, explaining a significant amount of BOLD signal variance in over 30% of pixels. Addition of the second order respiratory regressor (2R) and a single multiplicative term (11X) accounted for a small, but significant, additional amount of variance. Addition of cardiac terms and higher order respiratory and multiplicative regressors did not reduce BOLD signal variance by a significant amount. The optimized RETROICOR model (‘2R11X’) was used in the subsequent nested regression model analysis.

**Table 2.** *F*-test results showing the percentage of pixels within the fibroglandular tissue ROI where variance was significantly reduced by adding specific regressors, relative to the base model, averaged across subjects. Numbers in bold correspond to a significant percentage of pixels above a threshold set by the binomial (null) distribution (corresponding to a 0.01 false-positive rate). The optimal model for reducing variability in the BOLD signal is ‘2R11X’.

Regressors	Test model	Base model	% pixels
1C^a^	1C		1.1
1R^b^	1R		**30.7**
2C	2C1R	1C1R	1.0
2R	1C2R	1C1R	**1.5**
3C	3C2R	2C2R	0.7
3R	2C3R	2C2R	1.0
4C	4C3R	3C3R	1.2
4R	3C4R	3C3R	1.0
5C	5C4R	4C4R	0.8
5R	4C5R	4C4R	0.9
11X^c^	2C2R11X	2C2R	**1.9**
21X	2C2R21X	2C2R11X	1.3
12X	2C2R12X	2C2R11X	0.9
22X	2C2R22X	2C2R12X	1.0

a 1C: first order cardiac term (calculated according to equation (A.3) with *A*  =  1 and *B*  =  0).

b 1R: first order respiratory term (calculated according to equation (A.3) with *A*  =  0 and *B*  =  1).

c 11X: first order multiplicative term (calculated according to equation (A.4) with *C*_*m*_  =  1 and *D*_*m*_  =  1).

### 3.3. Impact of physiological corrections on signal variance

**Figure 3.**
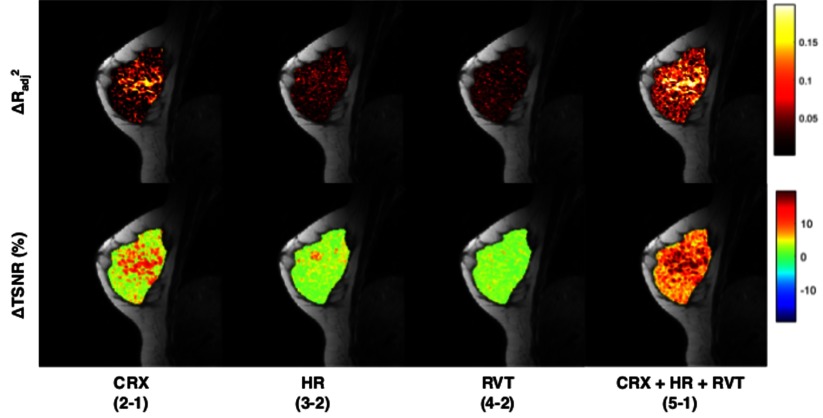
Maps showing successive differences in the adjusted coefficient of determination ($ \Delta R_{\text{adj}}^{2}$) between regression models and corresponding percentage difference in temporal signal-to-noise ratio (TSNR) of resting-state BOLD data, illustrating the importance of each set of physiological noise regressors in a representative volunteer. Maps are overlaid on an anatomical image with $ \Delta R_{\text{adj}}^{2}$ scaled between 0 and 0.2 and ΔTSNR scaled between  −20% and  +20%. The optimized RETROICOR model (denoted CRX) accounts for the largest amount of signal variance, corresponding to the brightest $ \Delta R_{\text{adj}}^{2}$ map for a single physiological noise model.

The impact of SyN-based non-rigid registration and the five physiological noise regression models on signal variance and image TSNR is summarized in table 3. A significant (*P*  <  0.001) increase (23.4%) in the mean TSNR of resting-state data was seen post-registration (comparing Models 0 and 1). The model comparison demonstrates the impact of each individual regressor in explaining additional variance, calculated pixel-wise within the ROI. After correction for low-frequency drifts attributed to hardware, adding the three optimal RETROICOR regressors (Model 2) accounted for the largest amount of additional variance in the resting-state BOLD signal ($ \Delta R_{\text{adj}(2-1)}^{2}$  =  3.3  ±  2.1%). Adding the measured HR and RVT regressors to Model 2 explained similar amounts of variance (2.0  ±  0.6% and 2.3  ±  0.3% respectively). The continued increase in mean $R_{\text{adj}}^{2}$ demonstrates that each of the regressors were useful in explaining additional variance in the data, up to a maximum of 16.4  ±  3.1% for Model 5. Corresponding significant increases (*P*  <  0.001) in the mean TSNR were also observed within the fibroglandular tissue ROI for each additional regressor, illustrated in figure 3 for a representative subject. Consistent with the variance analysis, RETROICOR had the largest impact on TSNR, increasing pixel-wise ΔTSNR by an average of 4.4  ±  1.4% across all subjects. ΔTSNR increased to 7.6  ±  1.5% when all physiological regressors were added to the model.

**Table 3.** Mean adjusted coefficient of determination ($R_{\text{adj}}^{2}$) and temporal signal-to-noise ratio (TSNR) of the resting-state BOLD signal within the fibroglandular tissue ROI, averaged across subjects for unregistered data and five nested regression models. Mean difference in voxel-wise $ \Delta R_{\text{adj}}^{2}$ and ΔTSNR (%) for each model comparison shows the effect of each correction. The optimized RETROICOR model (denoted CRX) accounts for the largest amount of signal variance (highest $ \Delta R_{\text{adj}}^{2}$ and %ΔTSNR).

Model	SyN^a^	Regressors				Mean $R_{\text{adj}}^{2}$	Mean TSNR	Model comparison	Mean $ \Delta R_{\text{adj}}^{2}$	Mean ΔTSNR (%)
		HW^b^	CRX^c^	HR^d^	RVT^e^					
		X				0.156 ± 0.114	35.0 ± 8.4			
1	X	X				0.091 ± 0.047	43.2 ± 10.8			
2	X	X	X			0.123 ± 0.035	44.9 ± 11.0	2-1	0.033 ± 0.021	4.4 ± 1.4
3	X	X	X	X		0.144 ± 0.031	45.6 ± 11.1	3-2	0.020 ± 0.006	1.5 ± 0.3
4	X	X	X		X	0.146 ± 0.034	45.6 ± 11.2	4-2	0.023 ± 0.003	1.6 ± 0.2
5	X	X	X	X	X	0.164 ± 0.031	46.2 ± 11.3	5-1	0.073 ± 0.024	7.6 ± 1.5

a Symmetric diffeomorphic normalization based non-rigid registration algorithm.

b Hardware (second order polynomial) regressor.

c Optimized RETROICOR model.

d Heart rate regressor.

e Respiratory volume per unit time regressor.

The SyN-based registration took approximately 6 min to align 180 images (using parallelization with four cores), whilst computational times for the GLM ranged from approximately 1 min (Model 1)–6.5 min (Model 5).

### 3.4. Impact of physiological corrections on BOLD parameter estimation

Non-rigid registration led to a significant reduction in the median correlation coefficient (*P*  =  0.033) and number of activated pixels (*P*  =  0.027) for resting-state data. The median correlation coefficient and area of activation also significantly decreased (*P*  =  0.003 and *P*  <  0.001) for the vasomotor stimulus. Overall, there was a significant difference in the median correlation coefficient and number of activated pixels between the resting-state and activated scans before any correction was applied (*P*  =  0.020 and *P*  =  0.015), which was improved after registration (*P*  =  0.012 and *P*  =  0.009). This difference was further improved by subtracting the three optimal RETROICOR regressors (‘2R11X’) from the registered data (*P*  =  0.002 and *P*  =  0.002).

**Figure 4.**
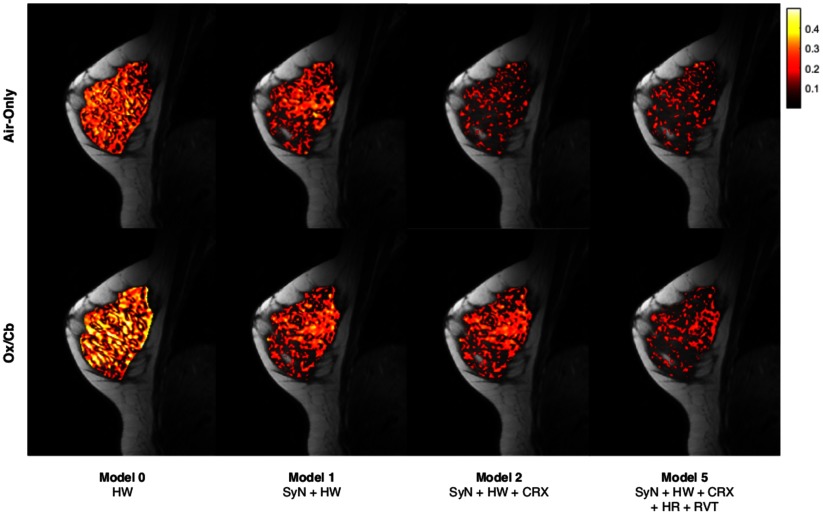
Activation maps showing magnitude of correlation coefficients (*P*  <  0.05) for a representative volunteer, illustrating the impact of physiological corrections on the detection of BOLD activation effects for air-only and oxygen-carbogen data. Maps are shown for the same subject as in figure 3. In this volunteer, SyN-based non-rigid registration reduced activation in both air-only and oxygen-carbogen states, addition of the optimized RETROICOR regressors (Model 2) removed false positive activations in the air-only state, whilst addition of HR and RVT regressors had a negative impact on activation detection during oxygen-carbogen gas breathing.

**Figure 5.**
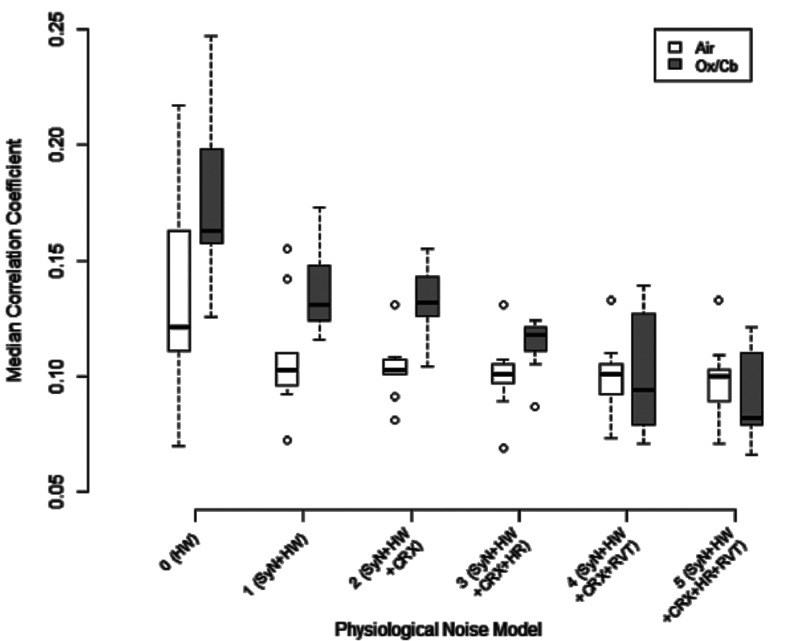
Boxplot showing the impact of SyN-based non-rigid registration and the five nested physiological noise models on the median correlation coefficient of air-only (resting-state) and oxygen-carbogen data. Over all volunteers, Model 2 (SyN  +  HW  +  CRX) yielded the most highly significant difference between air-only and oxygen-carbogen states.

Although regression of HR and RVT improved the TSNR of resting-state scans and significantly reduced the number of false-positive activations during air-only breathing (*P*  =  0.038, comparing Model 5 and Model 2), it also significantly reduced the median correlation coefficient and number of activated pixels (*P*  <  0.001) for the oxygen-carbogen stimulus paradigm, yielding no significant difference between resting-state and activated scans for Models 3–5. These results are summarized in table 4 and the impact of these corrections on detecting activation is illustrated in figure 4 for a representative subject. The median correlation coefficient for resting-state and oxygen-carbogen data is shown for all subjects and correction models in figure 5.

**Table 4.** Median correlation coefficient (CC) and percentage of activated pixels (*r*_m_  >  0.14) within the fibroglandular tissue ROI and statistical inferences comparing the oxygen-carbogen stimulus to resting-state (air-only) data, averaged across subjects for unregistered data and five physiological noise regression models.

Model	SyN	Regressors				Air-only		Oxygen-carbogen	
		HW	CRX	HR	RVT	Median CC	Activation (%)	Median CC (*P*-value)	Activation (%) (*P*-value)
		X				0.133 ± 0.046	39.3 ± 22.4	0.181 ± 0.047 (0.020)^a^	59.6 ± 13.5 (0.015)^a^
1	X	X				0.108 ± 0.025	27.4 ± 14.8	0.137 ± 0.020 (0.012)^a^	44.6 ± 9.8 (0.009)^a^
2	X	X	X			0.103 ± 0.013	24.5 ± 8.9	0.133 ± 0.016 (0.002)^b^	42.0 ± 9.3 (0.002)^b^
3	X	X	X	X		0.100 ± 0.016	23.2 ± 9.7	0.113 ± 0.012 (0.052)	31.6 ± 7.9 (0.038)^a^
4	X	X	X		X	0.100 ± 0.017	22.6 ± 10.6	0.104 ± 0.028 (0.702)	26.1 ± 16.7 (0.625)
5	X	X	X	X	X	0.098 ± 0.018	21.7 ± 11.0	0.092 ± 0.022 (0.594)	20.2 ± 12.7 (0.790)

a Significant *P*  <  0.05.

b Highly significant *P*  <  0.005.

## 4. Discussion

Signal intensity changes induced by the modulated gas stimuli are small, on the order of 1–2%, and the TSNR of the time series is critical to reliably detect vasomotor activation effects. Furthermore, the BOLD signal is dependent on various physiological parameters and it can be difficult to disentangle true vasomotor reactivity due to hypercapnic and hyperoxic gas from false activation effects that arise due to motion artefacts or natural physiological fluctuations influencing blood oxygenation. In this study we investigated the impact of various physiological correction models, both on the signal variance and TSNR, as well as activation parameters during air-only breathing (resting-state) and in response to a vasoactive stimulus design.

Respiratory effects were the dominant source of physiological noise in this study and registration of the dynamic series was important both in increasing TSNR and reducing false-positive activation effects, even when motion artefacts were small. After registration and correction for low-frequency hardware drifts, the optimized RETROICOR model accounted for the largest amount of additional signal variance. The subsequent increase in TSNR after application of RETROICOR translated to an improvement in BOLD sensitivity, demonstrated by the highly significant difference (*P*  =  0.002) between resting-state and vasoactive scans, illustrated in figure 4 (column 3). As the phase of respiration relative to the timing of image acquisition is expected to vary randomly, removing signal components that are correlated with the respiratory phase waveform intuitively should decrease signal variance and improve BOLD sensitivity. This approach could also be generalized to BOLD and *T*_1_-weighted oxygen-enhanced experiments investigating changes in oxygenation of healthy and diseased tissue, to improve detection of the small changes induced by the step change in inspired oxygen fraction. The performance of the optimized RETROICOR was not reported on unregistered data, as preliminary work demonstrated that applying the RETROICOR algorithm without registration did not perform as well (Wallace *et al* 2016b). Furthermore, in a study by Jones *et al* (2008) investigating the introduction of motion correction parameters into a RETROICOR-based regression paradigm in the brain, it was demonstrated that the optimal order of corrections in terms of temporal standard deviation reduction resulted by performing image registration prior to RETROICOR. This is expected, since the RETROICOR model does not account for movement of physiological fluctuations between voxels.

The ‘2R11X’ model was chosen as the optimal RETROICOR model as it accounted for significant variance in the data, without over-fitting to noise. The first order respiratory regressor accounted for the largest amount of structured noise in the BOLD signal. This regressor is likely to be correlated with the residual motion artefact or magnetic susceptibility changes that occur due to thoracic movement during respiration. Cardiac fluctuations did not account for a significant amount of noise in the data. In the brain, the magnitude of signal variation due to cardiac effects is often largest around major vessels (Dagli *et al* 1999, Glover *et al* 2000), so the absence of large vascular structures in the breast may help explain why the cardiac regressors were not useful in explaining BOLD signal variance in this case. The effect of including higher order respiratory or multiplicative harmonics was not found to be significant.

We also investigated the impact of including regressors describing low-frequency variation in heart rate and inspired respiratory volume per unit time, as these have been shown to be another source of signal variance in the fMRI literature. In agreement with previous studies in the brain, the HR and RVT regressors accounted for additional signal variance in the fibroglandular tissue ROI and led to incremental improvements in TSNR and a reduction of false-positive activation effects in the resting-state data (Shmueli *et al* 2007, Hutton *et al* 2011). Variations in the depth and rate of breathing will alter arterial CO_2_ levels. It has also been proposed that CO_2_-mediated vasodilation will in turn trigger chemoreflexes to adjust the depth and rate of subsequent breaths in order to maintain optimal blood gas parameters, thus forming a feedback cycle. The period of this chemoreflex-mediated feedback cycle has been measured between 25 s and several minutes, resulting in low frequency (<0.04 Hz) temporal fluctuations (Van Den Aardweg and Karemaker 2002). Therefore, accounting for subtle variations in heart rate and breathing patterns that occur naturally at rest intuitively will explain some of the variance in the BOLD signal and help account for false-positive activations.

Although increased TSNR should improve BOLD sensitivity, inclusion of lagged HR and RVT time courses in the regression model had a detrimental effect on the detection of vasomotor response to the vasoactive stimulus. This may be explained by the moderate correlations between the experimental design and these physiological time courses, evidenced by the peaks in the Fourier power spectra at the stimulus frequency. Kong *et al* (2012) similarly found that although regression of HR and RVT reduced BOLD signal variance, regression of these physiological time courses had a negative impact on parameter estimation in response to a painful thermal stimulus design due to concurrent changes in heart rate and breathing patterns. Without ground truth it is difficult to determine the relative impact of these processes, which may either remove false-positive activation effects or remove true active pixels that share variance with HR and RVT. In this case, the latter possibility is highly likely, given the established relationship between HR, RVT and blood CO_2_ levels, which is the source of the BOLD contrast being manipulated in this experiment. Therefore, although the combination of all regressors (Model 5) gave the largest improvement in TSNR, Model 2 gives the greatest improvement in BOLD sensitivity.

Several limitations are recognized in this work. First, we did not directly measure changes in end tidal CO_2_, which may have helped to disentangle BOLD reactivity to the inspired CO_2_ fraction from BOLD contrast changes due to fluctuations in RVT. Increased ventilation causes arterial oxygen saturation to increase on the order of ~1%, which would further reduce deoxyhaemoglobin levels in venous blood. In subjects where the correlation between RVT and BOLD response was particularly strong, it is possible that hyperventilation during carbogen breathing artificially increased the apparent reactivity to CO_2_; however, further work is needed to elucidate the exact mechanisms of BOLD response. Second, the temporal resolution of the BOLD acquisition used in this experiment (4 s/image) may not be able to sufficiently model cardiac noise fluctuations due to aliasing, which could provide an alternative explanation as to why inclusion of cardiac phase regressors did not have a significant impact on BOLD signal variability. Third, due to the nature of the GLM regression analysis, any shared variance between the physiological noise regressors and the BOLD signal will be removed, thereby reducing the detected BOLD activation for regressors correlated with the stimulus design. The cardiac and respiratory response functions derived in fMRI experiments were not applied here as they have not been found to be beneficial outside the brain (Kong *et al* 2012).

In conclusion, these results demonstrate that reducing signal variance attributed to physiological processes is associated with changes in activation parameter calculation, confirming the importance of certain physiological corrections in reliably detecting functional changes in the breast. The ‘2R11X’ RETROICOR model was found to be optimal in accounting for signal variance without over-fitting to noise and improved detection of BOLD activation effects. Subtle variations in HR and RVT that occurred naturally at rest accounted for additional variance in the resting-state BOLD data. However, inclusion of these regressors in the physiological noise model is not recommended as they appear to be correlated with the vasoactive stimulus design, making it difficult to disentangle BOLD signal reactivity due to CO_2_ changes from the associated ventilatory and cardiac responses.

## Figures and Tables

**Figure A1.**
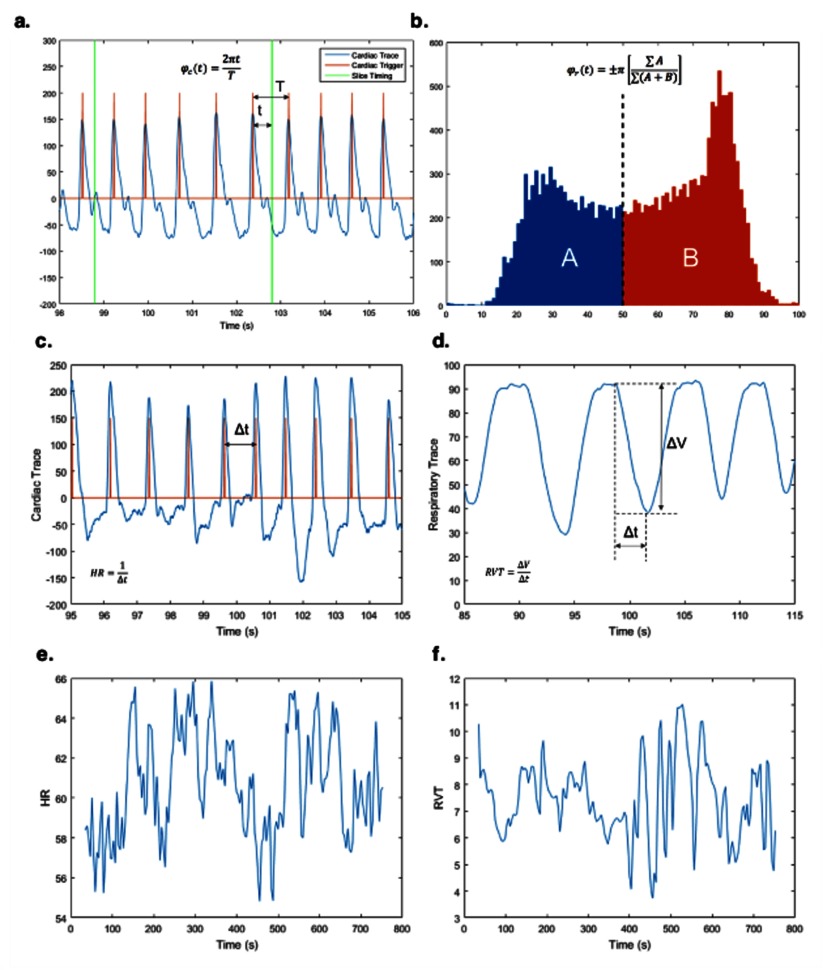
Schematic illustrations showing determination of physiolgoical noise regressors: calculation of (a) cardiac and (b) respiratory phases for RETROICOR; calculation of HR from the (c) cardiac time course and RVT from the (d) respiratory waveform; example (e) HR and (f) RVT time-courses extracted from physiological data.

## Appendix

### A.1. RETROICOR method

The RETROICOR correction method assumes that the functional time series in a voxel is corrupted by additive noise resulting from quasi-periodic cardiac and respiratory processes. The cardiac phase (*ϕ*_c_) is defined as follows (Glover *et al* 2000):

A.1 \begin{eqnarray*}{{\phi}_{\text{c}}}(i)=2\pi \frac{t(i)-{{t}_{\text{s}}}\left(\,j\right)}{{{t}_{\text{s}}}\left(\,j+1\right)-{{t}_{\text{s}}}\left(\,j\right)}\end{eqnarray*}

where *i* represents the *i*th image, *t*(*i*) is the image acquisition time and *t*_s_(*j*) and *t*_s_(*j*  +  1) are the peaks defining the beginning and end of the *j*th cardiac cycle, as illustrated in figure A1(a). The phase of respiration (*ϕ*_r_) is defined by the depth of breathing at the time of image acquisition (*R*(*t*)), relative to a histogram *H*(*b*) (scaled from 1 to 100) of the respiration depth across the entire scan, illustrated in figure A1(b). The transfer function relating respiratory amplitude and phase is given by:

A.2 \begin{eqnarray*}{{\phi}_{\text{r}}}(t)=\pm \pi \frac{\underset{{}}{\overset{{}}{{\sum\nolimits_{b=1}^{\text{rnd}\left[R(t)/{{R}_{\max}}\right]}}}}\,H(b)}{\underset{{}}{\overset{{}}{{\sum\nolimits_{b=1}^{100}}}}H(b)}\end{eqnarray*}

The physiological noise component may be modelled as a low-order Fourier series expanded in terms of the cardiac and respiratory phases as follows (Harvey *et al* 2008):

A.3 \begin{eqnarray*}\begin{array}{c} {{y}_{\delta}}(t)=\underset{A=1}{\overset{{{N}_{A}}}{\sum}}\left[{{\beta}_{1A}}\cos \left(A{{\phi}_{\text{c}}}(t)\right)+{{\beta}_{2A}}\sin \left(A{{\phi}_{\text{c}}}(t)\right)\right] \\ +\underset{B=1}{\overset{{{N}_{B}}}{\sum}}\left[{{\beta}_{3B}}\cos \left(B{{\phi}_{\text{r}}}(t)\right)+{{\beta}_{4B}}\sin \left(B{{\phi}_{\text{r}}}(t)\right)\right]+\underset{m}{\sum}{{\psi}_{m}} \end{array}\end{eqnarray*}

where *N*_*A*_  ⩽  5, *N*_*B*_  ⩽  5 and *β* represents unknown amplitude coefficients. The multiplicative terms *ψ*_*m*_ are given by:

A.4 \begin{eqnarray*}\begin{array}{c} {{\psi}_{m}}={{\beta}_{5m}}\sin \left({{C}_{m}}{{\phi}_{\text{c}}}+~{{D}_{m}}{{\phi}_{\text{r}}}\right)~+~{{\beta}_{6m}}\cos \left({{C}_{m}}{{\phi}_{c}}+{{D}_{m}}{{\phi}_{\text{r}}}\right)~ \\ +{{\beta}_{7m}}\sin \left({{C}_{m}}{{\phi}_{\text{c}}}-{{D}_{m}}{{\phi}_{\text{r}}}\right)+{{\beta}_{8m}}\cos \left({{C}_{m}}{{\phi}_{\text{c}}}-{{D}_{m}}{{\phi}_{\text{r}}}\right) \end{array}\end{eqnarray*}

where *C*_*m*_ and *D*_*m*_ are positive integers and *β* represents unknown amplitude coefficients.

A basis set of up to fifth order sine and cosine Fourier series components was calculated, based on the phase of the cardiac and respiratory cycles relative to the timing of each image acquisition, yielding a total of 20 regressors. An additional 16 sine and cosine regressors (i.e. *C*_*m*_ and *D*_*m*_  ⩽  2) were calculated to account for any interaction between these two processes.

This analysis was performed using a Matlab implementation of the modified RETROICOR algorithm, adapted from code developed by New York University Centre for Brain Imaging (cbi.nyu.edu/software). The code repository and sample physiological data is available at https://github.com/tesswallace/retroicor.

### A.2. Extraction of heart rate and respiratory volume per unit time

Heart rate was defined as the inverse of the interval between two consecutive beats, as illustrated in figure A1(c). Spurious beat frequencies were removed from the time course by replacing frequencies more than two standard deviations away from the local median with the median value within an 8 s sliding window. The resulting clean cardiac time course was averaged for each sliding window, defined by the (*k*  −  1)th, *k*th and (*k*  +  1)th TRs, yielding a measure of heart rate for each imaging time point, as shown in figure A1(e). The result was divided into 60 to convert to units of beats min^−1^.

A time series representing the percentage change in respiratory volume per unit time (RVT) was calculated from the normalized respiratory waveform as the difference between the maximum and minimum belt positions of each respiratory cycle (i.e. amplitude difference between inspiration and expiration), divided by the duration of respiration (i.e. the time between the peaks of inspiration and expiration), as illustrated in figure A1(d). The RVT time series was interpolated to the imaging TR to yield a measure for each time point, as shown in figure A1(f).

### A.3. Optimization of RETROICOR model

Each RETROICOR model was compared to a base model, containing the same set of regressors as the test model minus the regressor to be investigated. *F*-test regression was used to determine whether including higher order and multiplicative terms could explain a significant amount of additional variance in the BOLD signal. The number of pixels exceeding the threshold for the *F*-statistic (*P*  <  0.01) was counted for each subject. This was compared to a binomial null distribution parameterized by the total number of pixels in the ROI, which accounts for the fact that there will always be some random correlation with regressors. The null distribution threshold (*t*) for the number of significant *F*-test voxels was calculated such that:

A.5 \begin{eqnarray*}p\left(n~\geqslant ~t\right)=\underset{m=1}{\overset{N}{\sum}}\left(\begin{array}{c} N \\ m \end{array}\right){{p}^{m}}{{(1-p)}^{N-m}}~\leqslant ~0.01\end{eqnarray*}

Only regressors where the percentage of pixels with significantly reduced variance across all subjects exceeded this threshold (corresponding to a false positive rate of 0.01) were considered significant and were included in the final regression model.
